# Access, acceptability and utilization of community health workers using diagnostics for case management of fever in Ugandan children: a cross-sectional study

**DOI:** 10.1186/1475-2875-11-121

**Published:** 2012-05-24

**Authors:** David Mukanga, James K Tibenderana, Stefan Peterson, George W Pariyo, Juliet Kiguli, Peter Waiswa, Rebecca Babirye, Godfrey Ojiambo, Simon Kasasa, Franco Pagnoni, Karin Kallander

**Affiliations:** 1 Department of Epidemiology and Biostatistics, Makerere University School of Public Health, P.O. Box 7072, Kampala, Uganda; 2 Division of Global Health, IHCAR, Department of Public Health Sciences, Karolinska Institutet, Stockholm, SE, 17177, Sweden; 3 The African Field Epidemiology Network, P.O. Box 12874, Kampala, Uganda; 4 Malaria Consortium Africa, P.O. Box 8045, Kampala, Uganda; 5 International Maternal and Child Health Unit, Department of Women’s and Children’s Health, Uppsala University, Uppsala, Sweden; 6 Department of Health Policy, Planning and Management, Makerere University School of Public Health, P.O. Box 7072, Kampala, Uganda; 7 Department of Community Health and Behavioral Sciences, Makerere University School of Public Health, P.O. Box 7072, Kampala, Uganda; 8 Iganga/Mayuge Demographic Surveillance Site, P.O. Box 111, Iganga, Uganda; 9 Evidence for Antimalarial Policy and Access Unit, UNICEF/UNDP/World Bank/WHO Special Programme for Research and Training in Tropical Diseases (TDR), Geneva, Switzerland

**Keywords:** Community health worker, Case management, Malaria, Pneumonia, Febrile children, Diagnostics, Access, Acceptability, Utilization, Uganda

## Abstract

**Background:**

Use of diagnostics in integrated community case management (iCCM) of fever is recognized as an important step in improving rational use of drugs and quality of care for febrile under-five children. This study assessed household access, acceptability and utilization of community health workers (CHWs) trained and provided with malaria rapid diagnostic tests (RDTs) and respiratory rate timers (RRTs) to practice iCCM.

**Methods:**

A total of 423 households with under-five children were enrolled into the study in Iganga district, Uganda. Households were selected from seven villages in Namungalwe sub-county using probability proportionate to size sampling. A semi-structured questionnaire was administered to caregivers in selected households. Data were entered into Epidata statistical software, and analysed using SPSS Statistics 17.0, and STATA version 10.

**Results:**

Most (86%, 365/423) households resided within a kilometre of a CHW’s home, compared to 26% (111/423) residing within 1 km of a health facility (p < 0.001). The median walking time by caregivers to a CHW was 10 minutes (IQR 5–20). The first option for care for febrile children in the month preceding the survey was CHWs (40%, 242/601), followed by drug shops (33%, 196/601).

Fifty-seven percent (243/423) of caregivers took their febrile children to a CHW at least once in the three month period preceding the survey. Households located 1–3 km from a health facility were 72% (AOR 1.72; 95% CI 1.11–2.68) more likely to utilize CHW services compared to households within 1 km of a health facility. Households located 1–3 km from a CHW were 81% (AOR 0.19; 95% CI 0.10–0.36) less likely to utilize CHW services compared to those households residing within 1 km of a CHW.

A majority (79%, 336/423) of respondents thought CHWs services were better with RDTs, and 89% (375/423) approved CHWs’ continued use of RDTs. Eighty-six percent (209/243) of respondents who visited a CHW thought RRTs were useful.

**Conclusion:**

ICCM with diagnostics is acceptable, increases access, and is the first choice for caregivers of febrile children. More than half of caregivers of febrile children utilized CHW services over a three-month period. However, one-third of caregivers used drug shops in spite of the presence of CHWs.

## Background

Malaria and pneumonia are leading causes of morbidity and mortality among under-fives in sub-Saharan Africa [1-3]. Many of these deaths occur at home due to poor access to health care [1,4]. Community case management (CCM) of malaria and pneumonia have both been shown to reduce under-five mortality [5,6], and are recommended by the WHO [7-9]. Since 2002, Uganda has adopted and implemented CCM for malaria (locally referred to as home-based management of fever, HBMF). Under CCM, Community Health Workers (CHWs) provide pre-packaged anti-malarial drugs (initially chloroquine and sulphadoxine/pyrimethamine, and later artemisinin-based combination therapy - ACT) presumptively to children that present with, or have a history of fever [10]. In mid 2010 (after this study had commenced), Uganda adopted a national policy on integrated community case management (iCCM) for malaria, pneumonia and diarrhoea [11].

Parasitological confirmation in all patients presenting with symptoms compatible with malaria at all levels of the health system before administration of anti-malarial treatment has been recently recommended by WHO [12]. Parasitological confirmation improves quality of care and is particularly important in the context of declining malaria transmission, when malaria will be responsible for a decreasing proportion of fever cases [13].

Given that rapid diagnostic tests (RDTs) are now available with sensitivities comparable to routine microscopy in detecting malaria [14-17] these tests could improve diagnosis and quality of care of febrile children in malarious areas [18]. Use of diagnostic tools for malaria has the potential to reduction waste and cost of anti-malarial medication [19], potentially delay the development of parasite resistance to drugs, and improve treatment of alternative causes of fever such as pneumonia [20-22]. Indeed studies from Tanzania [23] and Zambia [24] have demonstrated decreased ACT prescription following use of RDTs by CHWs.

Respiratory rate timers (RRTs) [25] have been recommended by WHO and UNICEF as a diagnostic tool for pneumonia. The use of RRTs by CHWs in sub-Saharan Africa has been reported elsewhere [24,26].

A multi-country study [27] that included Uganda deployed and evaluated the feasibility of introducing RDTs and RRTs as diagnostic tools in iCCM. Prior to the study, a qualitative assessment of community acceptability of RDT use by CHWs was conducted in Uganda [28]. There is limited evidence of community access to, acceptability and utilization of programs deploying diagnostics into iCCM. Following one year of use of RDTs and RRTs by CHWs in iCCM, a follow up quantitative household survey was conducted to assess household access, utilization and acceptability of the use of RDTs and RRTs by CHWs. Questions about acceptability were informed by the findings of the qualitative study [28]. This paper reports key findings from this survey.

## Methods

### Study area

The study was conducted in the rural Ugandan district of Iganga as part of a larger study on the feasibility of deploying RDTs and RRTs at community level (Clinical Trials.gov Identifier NCT00720811). Uganda has an estimated population of 34 million inhabitants of whom about 80% live in rural areas. The district is located in South Eastern Uganda, approximately 112 km from Kampala, the capital city. It borders Mayuge district to the south, Bugiri to the southeast, Kaliro and Namutumba to the North and Jinja District to the West. The district is divided into three counties with a total of 19 sub-counties [29]. The total population of the district is approximately 600,000 and is mainly rural and engaged in subsistence agriculture. The district has one hospital, 23 HC IIIs, and 52 facilities providing only outpatient care. Iganga has high transmission rates (holoendemic) for malaria [30]. The study was conducted in Namungalwe sub-county which is comprised of seven parishes and 19 villages with a projected total population of 32,911. A total of 14 villages were selected to participate in the larger trial with half of them randomized into the intervention arm. This study was conducted in the seven intervention villages. In the intervention arm, CHWs were trained to use RRTs and RDTs to assess and manage febrile children as reported elsewhere [26].

### Study design and population

A cross-sectional household survey was conducted among caregivers of children 4–59 months old (under-fives). The study population consisted of caregivers (parents or guardians of children), and all under-five year old children within selected households. A caregiver was defined as any person above 18 years of age who at the time of the study was directly responsible for the care of an under-five eligible for this study, including seeking health services. They should have been responsible for the under-five for at least the preceding three months to the survey.

A household was defined as a group of people at the time of the study that lived together and ate from the same cooking pot. Polygamy is a common practice in the area, and for avoidance of doubt, in homesteads where different groups of people prepared and ate meals separately, each of these groups was considered a separate household. Households that had recently (no longer than three months) moved into the study area, child headed households, and households without under-fives were excluded from the study.

### Sample size consideration

A total of 423 households were selected to participate in the survey. The sample size was estimated using the formula for a single proportion by Kish [31], allowing for a sampling error of 6%, 95% confidence level, utilization of CHW services (primary outcome) of 50% and a design effect of 1.5. A non-response rate of 5% was allowed for.

### Sampling strategy

A household register was obtained from the Local Council I (village) chairperson, and updated with the help of the chairperson and a village scout. The updated total number of households in the area was 857 with Bufuntula village having 305, Nabikoote 154, Bubogo A 122, Namunkesu 114, Bubogo B 63, Namufuma 55, and Namunsala 44. The register was used as the sampling frame, and from this, the required number of households for the survey (as determined by probability proportionate to size sampling (PPS)) [32] was selected using a table of random numbers.

The household register(s) maintained by the chairperson record households using the name of head of household. In certain homesteads under a single head of household, there were more than one eligible household per operational definition. Within these eligible homesteads one household (per operational definition) was randomly selected using the ballot method.

Within selected households, all under-fives were included in the study. If a selected household was not eligible for the study, or declined the interview, the field team coordinator was contacted by the research assistant(s) via phone to provide a new household from the sampling frame. This was repeated for each village until the required sample per village had been enrolled. The non-response rate in this study was 3% (14 households).

### Data collection methods

A semi-structured questionnaire was used to collect data from the caregivers. Data were collected by a team of seven research assistants with experience in quantitative data collection drawn from the Makerere University Iganga/Mayuge Demographic Surveillance Site. Research assistants were trained for one day in the survey methods for this study, how to replace ineligible households in the field, and completing the study questionnaire. The research assistants participated in the pilot testing of the questionnaire in households that were not part of the pre-selected sample of households. Lessons from the pilot were used to revise the questionnaire to address issues of clarity and the logical flow of questions.

Data collection was over a 10-day period in the month of October 2010. Questions asked (or observations made) included: distance of the household from nearest health facility and CHW, socio-demographic characteristics of the head of household, type of housing (as a proxy to socio-economic status), history of fever among under-fives, health-seeking behaviour, perceptions of quality of services, utilization of CHW services, and perceptions of CHW services. The questionnaire can be obtained from the authors upon request.

Recall periods of one and three months were used in this study. The one-month recall was used for purposes of eliciting responses to questions about the management of the most recent fever episode in the under-fives. This was done to minimize recall bias because of the details expected from the respondents. The recall period was extended to three months for more general questions around health seeking choices made when an under-five had fever, reasons for choices, acceptability of diagnostics, as well as estimating utilization of CHW services, the primary outcome of this study. The extension was also driven by the fact that there had been a recent stock out of drugs among CHWs, that could have affected utilization and, therefore, a shorter recall period could result into an inaccurate estimate of utilization.

### Data management and analysis

Data was field edited for completeness and consistency by members of the study team, and changes were made in the field. Data were double entered into EpiData (EpiData Association, Odense, Denmark) statistical software, and analyzed using a combination of SPSS Statistics 17.0 (SPSS Inc) and Stata version 10 (College Station, Texas, USA). Data was checked for consistency, and cleaned.

The primary outcome (utilization of CHWs services) was estimated using the first healthcare option by caregivers of febrile children. Secondary outcomes included: access to CHW services, caregiver reported treatment outcomes for CHW services, proportion of caregivers who approved the use of RDTs by CHWs, reasons for non-acceptance of use of RDTs by CHWs, and perceptions about RRTs. Analysis adjusted for the clustering effect. Multivariate logistic regression analysis was used to explain the relationship between outcomes, and household as well as caregiver characteristics. A social economic status (SES) index was calculated for all households using materials used for the house roof, wall and floor. Principal components analysis (PCA) was used in constructing the index. Households were divided into five wealth quintiles based on their SES score [33]. The household quintile was taken as surrogate indicator for income. Odds Ratios with their corresponding 95% confidence intervals were generated.

The SES index was constructed as described by Vyas and Kumaranayake [33]. Materials used to construct floor, wall, and roof were used as a proxy. A total of 8 dummy variables were constructed. STATA version 10 was used to generate weights for each of the variables. These weights were then multiplied with the dummy scores for each individual household and added to generate a score for each household.

### Ethical approval

Ethical approval for the study was obtained from the World Health Organization Ethical Review Committee, Makerere University School of Public Health Institutional Review Board, and the Uganda National Council for Science and Technology. Permission was obtained from the Iganga District Health Office, and from the local authorities to conduct the study. Individual written informed consent was obtained from each of the caregivers. Confidentiality was maintained throughout data collection, management, analysis, and reporting.

## Results

### Background characteristics of households

Most heads of household were male (89.8%; 380/423), married (91.3%; 386/423), farmers (49.2%; 208/423) and had attained at least primary education (93.4%; 395/423) (Table 1). Median age of head of households was 39 years (IQR 32–47). There was slight female predominance (52.7%, 401/761) among the under-fives. Most (63.6%, 484/761) respondents were mothers of the under-fives.

**Table 1:** Background characteristics of households and under-fives

**Variable**	**Frequency (n = 423)**	**Percentage**
Location of households by village		
Bufutula	151	35.7
Nabikoote	76	18.0
Bobogo A	60	14.2
Namunkesu	56	13.2
Bubogo B	31	7.3
Namufuma	27	6.4
Namunsala	22	5.2
		
Head of household		
Sex		
Male	380	89.8
Female	43	10.2
Marital status		
Married/cohabiting	386	91.3
Widowed	23	5.4
Divorced/separated	11	2.6
Single	3	0.7
Religion		
Muslim	216	51.1
Protestant/Evangelical	172	40.7
Catholic	26	6.1
Other	4	0.9
Missing	5	1.2
Occupation		
Employed	34	8.0
Trader/self employed	176	41.6
Farmer	208	49.2
Does not work	5	1.2
Highest education level attained		
Never been to school	28	6.6
Primary	271	64.1
Secondary and above	124	29.3
		
Under-fives	(n = 761)	
Median age	36 months	IQR 19–48
Sex		
Male	360	47.3
Female	401	52.7
Relationship of respondent with under-5		
Father	138	18.1
Mother	484	63.6
Grandparent	90	11.8
Other	49	6.4
Fever in last 1 month		
Yes	601	79.0
No	160	21.0

Most main family houses were built with iron sheet roofs (68.8%, 291/423), were made of brick and cement walls (33.3%, 141/423), and had a mud floor (53%, 224/432).

Using the type of material used to build the main family house as a proxy for income, grass roofs, mud walls and mud floors were common at the lower end of the index, while iron sheets, plastered walls and cemented floors were common at the upper end of the distribution (Table 2). The distribution of households across different quintiles was as follows: Q1 (21.3%), Q2 (24.1), Q3 (26.7%), Q4 (18.7%), and Q5 (9.2).

**Table 2:** Composition of unique assets values

		**Type of materials used on the main house**		
Closest to	Index value (score)	Roof	Wall	Floor
Lowest value	−4.275	Grass	Mud	Mud
25th pecentile	−0.648	Iron sheets	Bricks/Mud	Mud
Median	0.150	Iron sheets	Bricks/cement	Mud
75th pecentile	1.417	Iron sheets	Bricks/Mud	Cement
Highest value	2.539	Iron sheets	Plastered cement	Cement

### Geographical access to services provided by community health workers

Eighty-six percent (365/423) of households reported to reside within a kilometre of a CHW’s home, compared with only 26.2% (111/423) residing within 1 km of a health facility (p < 0.001). Most (69.7%; 295/423) households reported to reside 1–3 km of a health facility. The median time it takes for households to walk to a CHW was 10 minutes (IQR 5–20).

### Utilization of services provided by community health workers

Fever in the last one month was reported in 79% (601/761) of all the children. More than half of children (380/601) were reported to have had more than one fever episode in the month preceding the survey. Respondents were asked about their first action for the most recent fever episode for each child in the month preceding the survey. Forty percent (242/601) sought care from a CHW, 32.7% (196/601) from a drug shop, 19.1% (115/601) from a health centre, 2.5% (15/601) from a hospital, 2.0% (12/601) gave drugs available at home, 0.3% (2/601) did nothing, while 3.2% (19/601) used other alternatives.

Respondents were asked about reasons for choice of first healthcare option. Fifty-one percent (305/601) mentioned convenient location, 30.3% (182/601) mentioned technical skills of personnel, 25.8% (155/601) were recommended by a friend/family, 10.5% (63/601) mentioned relatively low cost, 9.3% (56/601) mentioned courtesy of personnel, and 20.3% (122/601) had other reasons. The key “other” reasons were: CHW has drugs (62/122), and CHW was absent when I went there (29/122).

### Perceived quality of service

Ninety five percent (568/601) of respondents reported that they were satisfied with the services provided by the first option of care for the most recent fever episode. The aspects of services respondents were satisfied with were: drugs given (89.4%; 508/568), RDTs (33.5%; 190/568), RRTs (27.6%, 157/568), physical examination (18.5%, 105/568), laboratory tests (14.8%, 84/568), taking history (14.6%, 83/568), and others (2.6%; 15/568).

Of the 242 respondents whose first point of care was a CHW, 97.9% (237/242) reported that they were satisfied with the service they received. The main reasons for satisfaction were: availability of drugs (89.5%; 212/237), use of RDT (73.4%, 175/237), and use of RRT (60.8%, 144/237). Others were: the way the child was examined (20.3%, 48/237) and the way history was taken (9.3%, 22/237). Multiple responses were allowed for these questions on perceived quality.

### Utilization of CHW services in the three months preceding the survey and RDT acceptability

Table 3 shows the utilization of CHW services in the three months preceding the survey, as well as caregiver perceptions regarding use of malaria RDTs by CHWs. Fifty-seven percent (243/423) of respondents took an under-five to a CHW in the three month period preceding the survey. Reasons for not going to a CHW were: CHW had no drugs or was told they had no drugs (27.2%, 49/180); no child had been sick (21.7%; 39/180); do not like CHW services (12.7%; 23/180); did not know CHWs existed or where to find them (8.3% 15/119); CHWs was unavailable when they went there (7.2%; 13/180); CHWs are far compared to health centre (5.5%, 10/119), and a variety of other reasons (31/180).

**Table 3:** Utilization of CHW services and caregiver perceptions about CHW use of RDTs

Variable	Frequency (n = 423)	Percentage
Respondent visited CHW in past 3 months to seek care for under-five with fever		
Yes	243	57.4
No	180	42.6
		
Those who visited CHW	n = 243	
Was CHW using RDT		
Yes	212	87.2
No	28	11.5
Missing	3	1.2
Was CHW using RRT		
Yes	207	85.2
No	36	14.8
Was RRT useful		
Yes	209	86.0
No	29	11.9
Missing	5	2.1
		
Acceptability of RDTs (all respondents asked question regardless of visit to CHW in 3 months)	n = 423	
CHW services compared with other health services for children with fever		
Better	280	66.2
No difference	57	13.4
Worse	5	1.2
I don’t know	75	17.7
Other	4	0.9
Missing	2	0.5
CHW services before and after RDTs were introduced		
Now better	336	79.4
No difference	5	1.2
Worse	5	1.2
I don’t know	77	18.2
Should CHWs continue using RDTs		
Yes	375	88.7
No	1	0.2
Can’t answer the question	47	11.1

Of the respondents reporting to have visited a CHW in the three months preceding the survey, 87.2% (212/243) reported CHW was using RDTs. Sixty-six percent (280/423) of respondents thought CHW services were better than other health services for febrile children. Among those who visited a CHW in the three months preceding the survey 88.1% (214/243) thought CHW services were better than other health services.

Respondents were asked to compare CHWs services before and after introduction of RDTs. Seventy-nine percent (336/423) of all respondents, and 97.1% (236/243) of those who visited a CHW thought they are now better. Eighty-nine percent (375/423) of all respondents and 98.8% (240/243) who had visited a CHW would like CHWs to continue using RDTs.

A majority (99.1%; 419/423) of respondents said they had no fears or concerns regarding drawing of blood from children by CHWs. Among the four respondents who had fears, the reasons were: the agony of pain suffered by the child, and concerns about CHWs’ safe use of RDTs without causing infections.

### Adherence to test results for prescription of medicines

Of the 243 respondents who took a child to a CHW in the three months preceding the survey, 88.9% (216/243) said CHWs administered drugs based on test results, 7.8% (19/243) said “No”, while 3.3% (8/243) did not know. Among those who said “No”, the most commonly mentioned reason was that the CHW did not do the test, but gave the drugs anyway.

Respondents were asked if they trusted the test results, and 94.7% (230/243) said they did, while 5.3% (13/243) did not. Among those who said “No”, the most commonly mentioned reasons were: CHW did not do the test (6/13); CHW did not explain results (3/13); and, “my child did not improve” (2/13).

### Do caregivers influence CHW to administer drugs even when tests are negative?

A total of 41 respondents who visited the CHW in the three months preceding the survey reported to have recognized that one of the test results was negative. Of these, 12.2% (5/41) said they asked the CHW to administer medicines even when the result was negative. The reasons given for this were: “I saw my child was very sick”, and “I needed the medicine for protection” (precaution, just in case the test was wrong).

### Relationship between household characteristics and utilization of CHW services

Table 4 shows the relationship between household characteristics and utilization of CHW services. Utilization of CHW services was associated with distance of household to the nearest health facility. Households residing 1-3 km from a health facility were 72% (AOR 1.72; 95% CI 1.11–2.68) more likely to utilize CHW services compared to households residing within 1 km of a health facility.

**Table 4:** Association between household characteristics and utilization of CHW services

Variable	Utilized CHW services		AOR (95% CI)	p-value	
	Yes n = 243	No n = 180		
Distance to nearest Health Centre				
<1 km	54	57	1.00	
1–3 km	183	112	1.72 (1.11–2.68)	0.015*
>3 km	6	11	0.56 (0.18–1.64)	0.304
Distance to nearest CHW				
<1 km	229	136	1.00	
1–3 km	14	44	0.19 (0.10–0.38)	<0.001*
Education of head of household				
Never	18	10	1.00	
Primary	168	103	0.91 (0.37–2.17)	0.812
Secondary and above	57	67	0.47 (0.19–1.19)	0.080
Occupation of head of household				
Employed/self employed	127	83	1.00	
Farmer	114	94	0.79 (0.53–1.19)	0.241
Other/Casual	2	3	0.44 (0.05–3.29)	0.391
Indicator for SES				
Poorest (quartile 1)	49	41	1.00	
Second (quartile 2)	55	48	0.96 (0.54–1.69)	0.884
Middle (quartile 3)	73	40	1.53 (0.87–2.69)	0.143
Fourth (quartile 4)	44	34	1.09 (0.59–1.98)	0.798
Richest (quartile 5)	22	17	1.08 (0.51–2.31)	0.837

Utilization of CHW services was also associated with distance of the household to the nearest CHW. Households residing within 1-3 km from a CHW were 81% (AOR 0.19; 95% CI 0.10–0.38) less likely to utilize CHW services compared to households residing within 1 km of a CHW. Other relationships were not statistically significant.

## Discussion

This study assessed community access, utilization and acceptability of the use of malaria rapid diagnostic tests (RDTs) and respiratory rate timers (RRTs) by CHWs following one year of implementation. Accessibility to CHWs was high, with majority of the households residing within one kilometer of a CHW’s home. Most respondents reported taking 10 minutes to walk to a CHW’s home. Utilization of CHW services for febrile children was high, with more than half of respondents reporting to have taken an under-five to a CHW in the three-month period preceding the survey. About 80% of all respondents reported CHWs services as better after introduction of RDTs, while among respondents that utilized CHWs services in the three months to the survey, nearly all reported services as having improved following introduction of RDTs. Majority of respondents reported that RRTs were useful. Utilization of CHW services was associated with distance of household to the nearest health facility, and distance of household to nearest CHW. Households residing 1-3 km from a health facility were 72% more likely to utilize CHW services compared to households residing within 1 km of a health facility. Households residing within 1-3 km from a CHW were 81% less likely to utilize CHW services compared to those households residing within 1 km of a CHW.

The high accessibility by households to CHWs suggests that the program is meeting its goal of bringing curative services for febrile children as close as possible to their homes. CHW programs have been reported to improve access to prompt treatment for febrile children [34-37].

CHWs were the preferred choice for care for febrile children with drug shops a close second at over 30%. Drug shops remain very popular in spite of available free services for febrile children through CHWs. There is extensive literature from Tanzania [38-41], Kenya [42,43], Uganda [44,45] and elsewhere showing the important role that drug shops play as a source of care especially for malaria. Some of the reasons why caregivers did not utilize CHW services were lack of drugs, dislike of CHW services, not being aware of the CHWs services in the community, missing CHW in their homes on a visit, or being closer to a health facility. The program indeed experienced drug stock-outs from time to time, and this appears to have had a significant impact on caregiver choices. In many cases, caregivers continued to bypass CHWs and go elsewhere even when drug stocks had been replenished. CHW programs need to take measures to ensure stock-outs do not occur, or are kept at a minimum. In case it becomes unavoidable, there should be clear and timely information regarding when drugs are expected. Distance to the provider, and perceived skills of the provider were also found to be key drivers of choice of service provider. CHW programs need to ensure that the majority of community members have easy reach to CHWs, especially those in rural and hard to reach areas. A systematic review of access and utilization of health services shows that availability of drugs, distance to health facilities, and perceived quality of care are the key determinants influencing health service utilization [46].

Geographical factors influenced utilization of CHW’s services. The closer caregivers were to CHWs the more likely they were to use them. This is consistent with the program objectives. However, caregivers who were close (within a kilometer) to a health facility were less likely to utilize CHWs compared to those who resided farther way from a health facility. This finding has policy implications for CHW programs such as this. Since programs are designed to provide access to care for under-served, hard-to-reach communities, CHWs will need to be located carefully, so that only under-served communities are selectively included. In well-served communities where CHWs exist, they could provide services that complement what a nearby facility provide, including health education, health promotion, and referral services. Programs in these areas will also need to take into account the other contextual factors such as buy-in from medicine sellers as proposed by Goodman *et al.*[47]. Lehmann *et al.*[48] have made the case that CHWs are neither a panacea, nor cheap option for weak health services. As health services increasingly get strengthened and coverage improves, the roles of CHW will need to be carefully defined and refocused. Equally important is the fact that even when services are available they may not be accessible to the poorest in a community and in turn enhance inequities [49]. CHWs have a role in bringing these services closer to the poorest and excluded segments of communities.

Majority of caregivers that visited a CHW were satisfied with the service they received. Availability of drugs and use of diagnostics in this setting were key drivers of satisfaction. This is consistent with findings reported by Nsabagasani *et al.*[50] from western Uganda where both CHWs and caregivers agreed that diagnostic equipment at community level would improve diagnosis and attract more caregivers of febrile children.

Caregivers do not want to go where there are no drugs, as they feel they are wasting time and will have to go to the next provider.

Almost all caregivers had no fears about drawing of blood from under-fives by CHWs for the RDT test. Similar results have been reported from Zambia in a study where CHWs used RDTs in home management of childhood fever [51]. An earlier qualitative study from this area reported that some caregivers expressed fear that the blood collected could be used for HIV testing, the procedure could infect children with HIV, and the blood could be used for witchcraft [28]. This study was conducted prior to the introduction of RDTs in the community. It appears that the direct interaction of caregivers with competent CHWs [26] using the RDTs, and the community engagement that was undertaken prior to the intervention may have changed some of the negative perceptions. Majority of caregivers thought CHW services were better after introduction of RDTs, and nearly 90% of all caregivers interviewed approved of CHWs continued use of RDTs and RRTs.

The overall RDT positivity rate in the intervention study preceding this study was 88% (857/975) [27]. Acceptability of RDTs by caregivers might be different in settings were the positivity rate is much lower. In situations where caregivers of children feel that their children are “ill enough” to warrant prescription of an anti-malarial drug, with or without a positive test result, and where CHWs strictly adhere to RDT test results to guide prescription practices, it is plausible that popularity of tests and CHWs will be lower. The importance of identifying alternative causes of fever will be even more critical in these settings, as well as ensuring that CHWs are equipped to manage conditions such as non-severe pneumonia as has been reported elsewhere [24,27]. A study from the Solomon Islands, on acceptability of RDTs reports a general distrust by the community of the accuracy of RDTs, resulting in continued presumptive treatment of malaria [52]. Also, a study from Sudan reports that although the use of RDTs seemed to have improved the level of accuracy and trust in the diagnosis, 30% of volunteers did not rely on the negative RDT results when treating fever cases [35]. These mistrusts may be a result of the lack of intensive CHW training and supervision, as well as absence of services for alternative causes of fever at the CHW’s post.

Respondents reported that the majority of CHWs adhered to test results for prescription of drugs to patients. A small portion of CHWs were reported to have been coerced into providing medication to children with a negative RDT or RRT. This is in contrast to a study from Zambia that found adherence to test results to be high with over 99% of patients with a negative RDT result not prescribed an anti-malarial drug [51].

### Methodological considerations

Bias in recall was a potential problem in this study with caregivers being asked to remember things that happened in the past about an event that may not have been a major event in the home. This was minimized by limiting the recall period to one month for key details, and three months for more general questions about a most recent fever episode in the child. Caregivers responsible for under-fives and not anyone in the household, were interviewed.

Respondents were asked to compare current CHW services with those before introduction of RDTs. It was not established who had utilized CHWs before introduction of RDTs and it is likely that some respondents had never used CHWs in the areas before RDT introduction, but nevertheless responded to the question. This could lead to over or under estimation of the true positive or negative responses.

Interviewer bias is always a possibility in these kinds of studies. This was minimized by using research assistants who had not been involved in the intervention and were blinded to the hypothesis the data collection was based on.

Principal components analysis was used to generate a socio-economic status (SES) index. Fewer variables were used for this study than most often used, for example in the 2006 Uganda Demographic and Health Survey [53]. However, the index does portray the actual picture on the ground in terms of the components used and what is generally understood as what these components represent in terms of SES in the community. While asset-based measures are increasingly being used, there continues to be some debate about their use. These measures are more reflective of longer-run household wealth or living standards, failing to take account of short-run or temporary interruptions, or shocks to the household [54]. Therefore, if the outcome of interest is associated with current resources available to the household (as health services utilization might be), then an index based on assets may not be the appropriate measure [33]. However, under the circumstance, with no other source of information on household income and expenditure in this rural community with a large informal sector, the approach used was considered a reasonable alternative.

## Conclusion

ICCM with diagnostics is acceptable, increases access, and is the first choice for caregivers of febrile children. However, one-third of caregivers used drug shops in spite of the presence of CHWs. This implies that the service still needs to be better known, and better accepted, and CHWs need to have a constant supply of commodities. It also underscores the significant role played by drug shops and the need to involve them during programming. More than half of caregivers of febrile children utilized CHW services over a three-month period. It appears that the use of RDTs and RRTs may have improved the utilization of CHWs services.

## Competing interests

The authors declare that they have no competing interests.

## Authors’ contributions

DM, JKT, SP, GP, JK, PW, FB, RB, GO, FP and KK took part in designing the study, in tools development, in data analysis and in manuscript writing. SK participated in data analysis and manuscript writing. DM, RB, and GO participated in data collection. All authors approved the final manuscript.
